# Aquaporin‐4 activation facilitates glymphatic system function and hematoma clearance post‐intracerebral hemorrhage

**DOI:** 10.1002/glia.24639

**Published:** 2024-11-12

**Authors:** Wenchao Chen, Chuntian Liang, Shasha Peng, Shuangjin Bao, Fang Xue, Xia Lian, Yinghong Liu, Gaiqing Wang

**Affiliations:** ^1^ Department of Neurology Second Hospital of Shanxi Medical University, Shanxi Medical University Taiyuan Shanxi China; ^2^ Department of Neurology, Sanya Central Hospital (Hainan Third People's Hospital) Hainan Medical University Sanya Hainan China; ^3^ Department of Pharmacology, School of Basical Medical Sciences Shanxi Medical University Taiyuan China; ^4^ Department of Pharmacy, Sanya Central Hospital (Hainan Third People's Hospital) Hainan Medical University Sanya China; ^5^ Department of Pathology and Pathophysiology, Basic Medical College Shanxi Medical University Taiyuan China; ^6^ Department of Pathology West China Fourth Hospital Chengdu China

**Keywords:** AQP4, glymphatic system, hematoma clearance, intracerebral hemorrhage

## Abstract

Efficient clearance of hematomas is crucial for improving clinical outcomes in patients with intracerebral hemorrhage (ICH). The glymphatic system, facilitated by aquaporin‐4 (AQP4), plays a crucial role in cerebrospinal fluid (CSF) entry and metabolic waste clearance. This study examined the role of the glymphatic system in ICH pathology, with a focus on AQP4. Collagenase‐induced ICH models were established, with AQP4 expression regulated through mifepristone as an agonist, TGN‐020 as an inhibitor, and *Aqp4* gene knockout. Fluorescence tracing and multimodal magnetic resonance imaging (MRI) were employed to observe glymphatic system functionality, hematoma, and edema volumes. Neurological deficit scoring was performed using the modified Garcia Scale. AQP4 expression was quantified using RT‐qPCR and Western blotting, and cellular localization was explored using immunofluorescence. The brain tissue sections were examined for neuronal morphology, degenerative changes, and iron deposition. Three days post‐ICH, the AQP4 agonist group showed increased AQP4 protein expression and perivascular polarization, decreased hemoglobin levels, and reduced iron deposition. Conversely, the inhibition group exhibited contrasting trends. AQP4 activation improved glymphatic system function, leading to a wider distribution, improved neurological function, and reduced hematoma. Pharmacological inhibition and genetic knockout of AQP4 have opposing effects. The glymphatic system, facilitated by AQP4, plays a crucial role in hematoma clearance following cerebral hemorrhage. Upregulation of AQP4 improves glymphatic system function, facilitates hematoma clearance, and promotes brain tissue recovery.

## Main Points

- The glymphatic system aids hematoma clearance after intracerebral hemorrhage.
- The upregulation of AQP4 can enhance the glymphatic system, and vice versa.
- Findings suggest potential therapies for better outcomes in cerebral hemorrhage.

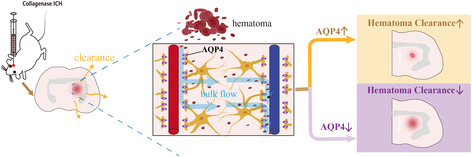

## 1 INTRODUCTION

Intracerebral hemorrhage (ICH) is a common subtype of stroke associated with adverse outcomes primarily linked to the formation of a hematoma (Li et al., 2020). Metabolic byproducts from hematoma, including hemoglobin (Hb), hemin, and iron, contribute to cerebral edema, blood–brain barrier (BBB) disruption, and neuronal death (Aronowski & Zhao, 2011). Enhancing endogenous brain clearance is crucial for improving ICH prognosis (Wang et al., 2018). The endogenous brain clearance system encompasses the vascular clearance system crossing the BBB, the phagocytic cell enzymatic degradation pathway, and brain lymphatic drainage systems, such as glymphatic and meningeal lymphatic systems (Liu et al., 2021; Zhao et al., 2007).

The earliest report of meningeal lymphatic vessels dates back to Mascagni's work (Bucchieri et al., 2015), with Andres later describing their structure using electron microscopy (Andres et al., 1987). The foundational concept of the glymphatic system was developed by Roy Weller and Roxana Carare (Weller et al., 1996), who highlighted the role of perivascular spaces in clearing interstitial solutes from the brain. In recent years, Nedergaard and her team introduced the term “glymphatic system,”(Iliff et al., 2012) and Louveau et al. subsequently confirmed the presence of lymphatic vessels in the dura mater using modern imaging techniques (Louveau et al., 2015), emphasizing the importance of brain lymphatic drainage in waste clearance.

The glymphatic system, formed by astrocyte processes around blood vessels, facilitates the entry of cerebrospinal fluid (CSF) into the parenchyma, exchanging with interstitial fluid (ISF) through aquaporin 4 (AQP4) water channels on the astrocyte end‐feet. This fluid ultimately drains through the meningeal lymphatic vessels into the cervical lymph nodes; it can also travel along the arachnoid sheath of the olfactory nerves, pass through the cribriform plate, and reach the submucosa of the nasal cavity, eventually entering the cervical lymph nodes. (Cserr & Knopf, 1992; Rasmussen et al., 2018; Weller et al., 1996). AQP4, expressed on astrocyte end‐feet, acts as a water gate, facilitating CSF entry for metabolite exchange (Rasmussen et al., 2018). High AQP4 expression around blood vessels (AQP4 polarization) promotes CSF flow in the brain (Hablitz et al., 2020). Inhibition or knockout of AQP4 reduces the clearance capability of the brain, emphasizing its crucial role in metabolite and toxin clearance (Zhao et al., 2017). Further exploration of AQP4's function and mechanisms in hematoma clearance post‐ICH is warranted.

In this study, we investigated the contribution of the glymphatic system to hematoma clearance and outcomes following ICH. Through pharmacological and genetic knockout approaches, we explored the effects of AQP4 interventions to identify changes in glymphatic system function. We assessed lymphatic clearance functionality in wild‐type C57 mice under normal and ICH conditions, discussed the impact of *Aqp4 gene* knockout on lymphatic clearance in ICH mice, and examined changes in glymphatic system function following treatment with the AQP4 agonist mifepristone (MFP) (Wei et al., 2019) and the inhibitor TGN‐020 (S et al., 2020) in ICH models.

The AQP4‐mediated glymphatic system has been shown to play a critical role in the clearance of Aβ and Tau proteins in Alzheimer's disease (Harrison et al., 2020; Peng et al., 2016), but its role in hematoma clearance following ICH has not yet been clearly elucidated. Therefore, this study aims to explore the role of AQP4 and the glymphatic system in hematoma clearance after ICH, providing new insights into post‐ICH recovery.

## 2 MATERIALS AND METHODS

This study was conducted and reported in accordance with the ARRIVE guidelines (Animal Research: Reporting of In Vivo Experiments) to ensure comprehensive and transparent reporting of animal research findings (C et al., 2010). All animal studies were conducted in accordance with the guidelines outlined in the National Institutes of Health Guide for the Care and Use of Laboratory Animals, as summarized in the Guidelines for Reporting In Vivo Experiments. Approval for the study was obtained from the Ethics Committee of the Second Clinical Medical College at Shanxi Medical University (Approval NO: DW2022052). In this study, 184 C57BL/6 (wild‐type) mice were obtained from the Animal Experiment Center of Shanxi Medical University, while 38 *Aqp4 gene* knockout mice (C57BL/6N‐*Aqp4* ^em1cyagen^) were sourced from Saier (Suzhou) Biotechnology Co., Ltd. Four collagenase‐induced ICH mice died post‐surgery due to subarachnoid hemorrhage (SAH), and they were excluded from statistical analysis. For humane considerations (weight loss >15% of preoperative weight), two mice were excluded from the final statistical analysis. Randomization, employing a random number table method, was utilized in all studies, with animals being randomly assigned to different groups. Behavioral tests and all other analyses were conducted by researchers who were unaware of the experimental groups and treatments.

We established an ICH model by injecting collagenase (Hablitz et al., 2020; Wang, Manaenko, et al., 2017). The experimental animals were divided into the sham operation group (SHAM), the ICH group, the ICH group treated with either the AQP4 agonist MFP or the AQP4 inhibitor TGN‐020, and the AQP4 knockout group. The drug intervention was administered 6 hours after establishing the ICH model. Neurological function deficits were assessed on days 1, 3, and 7 post‐ICH using the Modified Garcia Scale by collaborators who were blinded to the experimental groups and treatments.

On day 3 post‐ICH, after decapitation, the brain tissue was collected to measure brain water content, BBB permeability, and Hb levels. RNA and protein were extracted from the brain tissues of each group for quantitative PCR (qPCR) analysis of AQP4, and Western blot (WB) analysis was performed for quantification. After preparing frozen brain sections, Nissl staining, Fluoro‐Jade C (FJC) staining, and double immunofluorescence staining were performed to observe the co‐expression of AQP4 with blood vessels and astrocytes (Ishida et al., 2020; Nagelhus & Ottersen, 2013; Zhou et al., 2020). Iron staining was conducted on brain tissue sections collected on day 7 post‐ICH.

Additionally, fluorescent tracer detection was performed on brain tissue and lymph nodes on days 1, 3, and 7 post‐ICH. Prior to sacrificing the mice, FITC fluorescent tracer was injected, followed by sectioning of brain tissue and deep cervical lymph nodes. The distribution of the tracer was observed under a fluorescence microscope. Dynamic multimodal magnetic resonance imaging (MRI) was used for in vivo observation. The contrast agent Gd‐DTPA was injected into the hemorrhagic region, and the distribution of the contrast agent, ICH volume, and edema volume in the brains of mice in each group were dynamically monitored at different time points post‐ICH.

For detailed descriptions of materials and methods, please refer to the Supporting Information.

## 3 STATISTICAL ANALYSIS

Data statistical analyses were conducted using SPSS 20.0 and GraphPad Prism 9.3 software. Descriptive statistics for continuous variables were expressed as mean ± standard deviation. One‐way analysis of variance (ANOVA) was employed for comparisons among multiple groups, with post‐hoc Tukey tests for multiple comparisons between groups. A *p*‐value of less than 0.05 was considered statistically significant.

## 4 RESULTS

### 4.1 Impact of AQP4 on neurological damage, BBB permeability, Hb content, and brain water content after ICH

**FIGURE 1:**
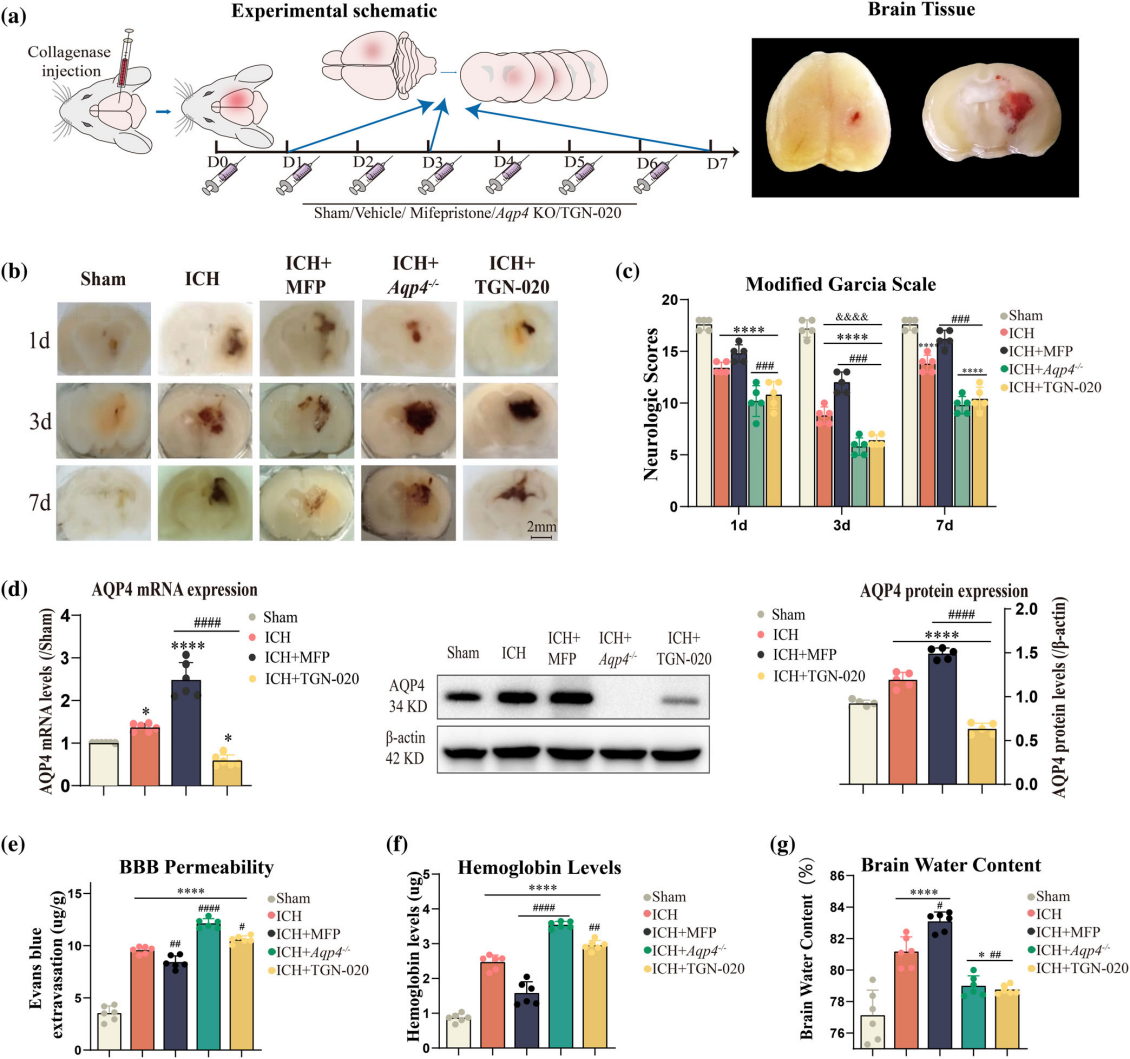
Impact of AQP4 on neurological damage, BBB permeability, hemoglobin content, and brain water content after ICH. (a) Experimental schematic. The brain tissue samples and coronal sections of brain tissue with hemorrhagic lesions were obtained. (b) Coronal section image of hematoma in mice brain after ICH. Black scale bar: 2 mm (c) Neurobehavioral assessments of mice at various time points in different groups. (*n* = 5). (d) Quantification of AQP4 mRNA and protein expression levels in each mouse group 3 days post‐ICH, along with representative Western blot (WB) images(*n* = 6). Comparison of blood–brain barrier permeability (e), hemoglobin levels (f), and brain water content (g) among different groups. (*n* = 6). *p < 0.05/****p < 0.0001 versus Sham group at the same time point. ^#^ *p* < 0.05/ ^##^ *p* < 0.01/ ^###^ *p* < 0.001/^####^ *p* < 0.0001 versus ICH group at the same time point. ^&&&&^ *p* < 0.0001 versus the same group at day 1 and day 7. AQP4, aquaporin‐4; ICH, intracerebral hemorrhage.

Following the described materials and methods, a mouse ICH model was established, and the resulting groups were as follows: Sham, ICH, ICH + MFP, ICH + *Aqp4* ^−/−^, and ICH + TNG‐020, then we obtained mouse brain tissue for testing (Figure 1a). From coronal sections of harvested mouse brain tissue, it was observed that at equivalent time points, the Sham group exhibited no hematoma, while the MFP intervention group showed smaller hematoma areas. Conversely, the TNG‐020 intervention and *Aqp4−/−* groups demonstrated larger hematoma areas. Within‐group comparisons, hematoma areas appeared larger at 3 days, with a trend towards improvement in bleeding conditions by day 7 (Figure 1b). Blind assessments using the Modified Garcia Scale at 1, 3, and 7 days revealed significant neurological impairment in the ICH group at all three time point compared to the Sham group (*p* < 0.0001, Figure 1c). On the third day, the ICH group was observed to exhibit a diminished neurological deficit score compared to both the first and seventh days (*p* < 0.0001, Figure 1c), indicating the most severe neurological deficit on the third day. Hence, we chose the third day following the most severe neurological deficit post‐ICH as the subsequent focal time point for our research. On the third day, between‐group comparisons indicated increased scores with MFP intervention (*p* < 0.0001) and decreased scores with TGN‐020(*p* = 0.0009) and *Aqp4* gene knockout (*p* < 0.0001), as compared to the ICH group(Figure 1c). Hematoma imaging disclosed larger areas in the 3‐day group than in the 1‐day and 7‐day groups.

In the following steps, we measured AQP4 levels in mouse brain tissues obtained 3 days after ICH. This was done to ensure the efficacy of our AQP4 intervention. The *Aqp4 gene knockout* group showed no detectable mRNA or protein expression. Both RT‐qPCR and WB results consistently demonstrated that the mRNA (*p* = 0.0418) and protein (*p* < 0.0001) expression levels of AQP4 in the ICH group were significantly higher than those in the Sham group, indicating an upregulation of AQP4 (Figure 1c). MFP administration further increased AQP4 expression, while significant decreases were observed in the TGN‐020 groups (*p* < 0.0001, Figure 1d).

Subsequently, we evaluated BBB permeability in mice 3 days post‐ICH. Compared to the Sham group, ICH significantly increased BBB permeability (*p* < 0.0001). The MFP group (*p* = 0.0055) exhibited reduced permeability, while the *Aqp4* ^*−/*^ (*p* < 0.0001) and the TGN‐020 (*p* = 0.0174) groups showed elevated permeability, with statistical significance (Figure 1e). These findings indicate that MFP intervention can ameliorate BBB disruption following ICH. Furthermore, we measured the Hb content in each group. The Hb levels were higher in the ICH group compared to the Sham group. In contrast with ICH, the MFP group (*p* < 0.0001) exhibited decreased Hb levels, while the *Aqp4* ^*−/−*^ (*p* < 0.0001) and the TGN‐020 (*p* = 0.003) groups displayed increased levels (Figure 1f). T. Finally, we assessed the brain water content in each group. Brain water content trends revealed increases in the ICH group compared to the Sham group (*p* < 0.0001). *Aqp4 gene* knockout (*p* = 0.004) and TGN‐020(*p* = 0.001) mediated AQP4 inhibition reduced brain water content compared to ICH, while MFP (*p* = 0.014) treatment increased it, with statistical significance (Figure 1g).

### 4.2 Neuronal morphological alterations and the accumulation of hemosiderin

**FIGURE 2:**
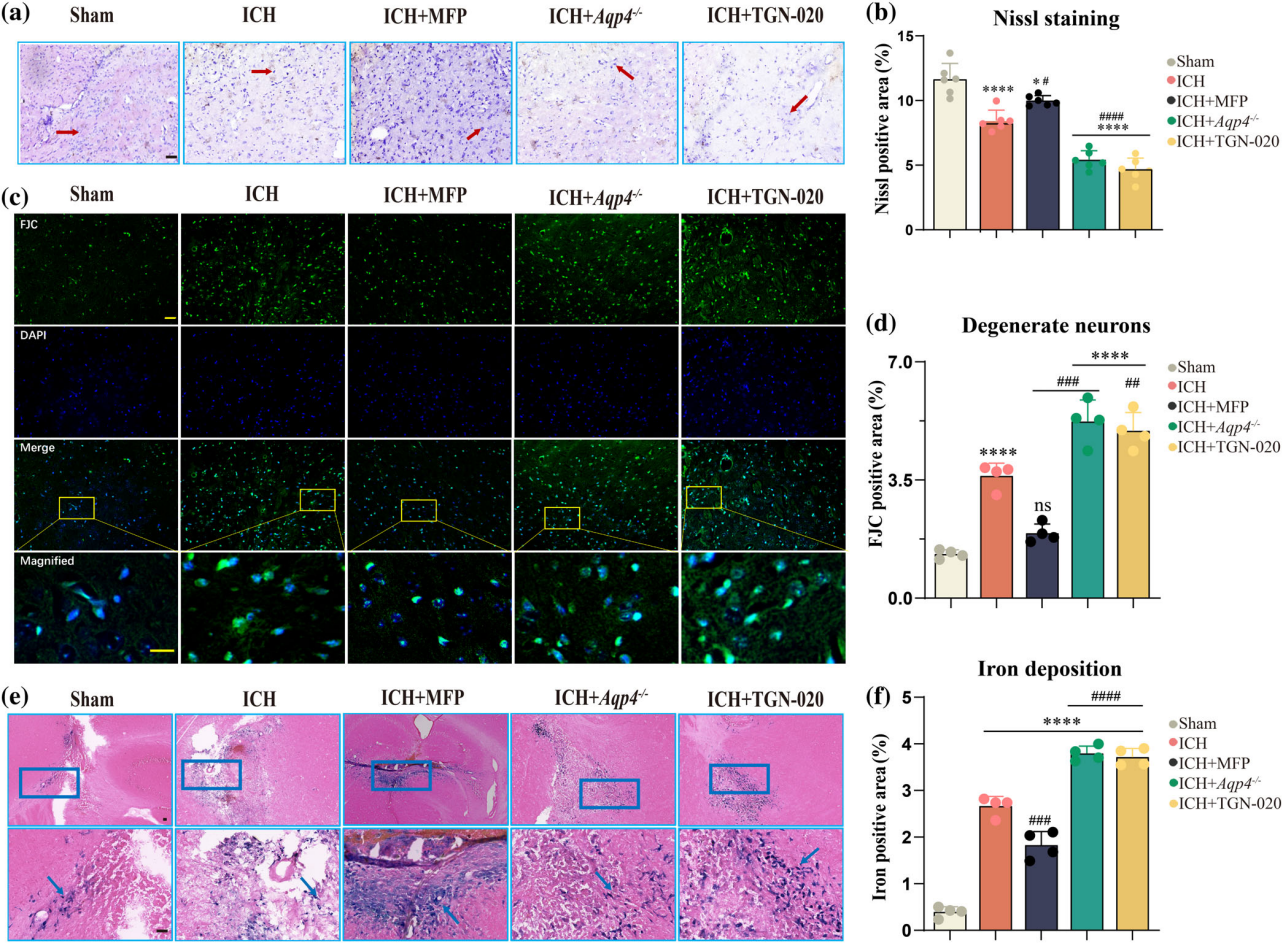
Influence of AQP4 on neuronal dynamics and iron deposition post‐ICH. (a)Representative images and (b) quantification of Nissl staining in brain tissue at 3 days post‐intracerebral hemorrhage (*n* = 6). The red arrows indicate normal neurons. Black scale bar:50 μm. (c) Representative images of FJC staining on 3 days after ICH. (d) Quantification of FJC staining around the hematoma (*n* = 4). Yellow scale bar:50 μm. (e) Representative brain sections stained with Perl's at day 7 post‐ICH in mice. Black scale bar:50 μm. (f) Quantification of iron deposition area at the hematoma core (*n* = 4). The blue arrows indicate iron deposition. ns: *p* > 0.05 versus Sham group. *p < 0.05/****p < 0.0001 versus Sham group. ^#^ *p* < 0.05/^###^ *p* < 0.001 /^####^ *p* < 0.0001 versus ICH group. AQP4, aquaporin‐4; ICH, intracerebral hemorrhage.

We used Nissl staining to examine the neuronal changes 3 days post‐ICH. The neurons in the Sham group had a higher count, dense arrangement, and prominent Nissl bodies. The ICH group showed partial Nissl body dissolution, lighter staining, and looser neuronal structure. The *Aqp4* ^*−/−*^ and TGN‐020 groups exhibited more pronounced damage (*p* < 0.0001, Figure 2a,b). MFP intervention improved Nissl staining compared to the ICH group, indicating partial neuronal injury amelioration (*p* = 0.043, Figure 2a,b). Similar trends were observed in FJC staining. All groups had increased FJC‐positive staining compared to the Sham group, with *Aqp4* ^*−/−*^ and TGN‐020 showing higher expression than the ICH group (*p* < 0.0001). MFP intervention reduced FJC‐positive expression (*p* < 0.0001, Figure 2c,d). Iron staining at 7 days post‐ICH showed increased hemosiderin deposition in all groups compared to the Sham, and was significantly higher in *Aqp4* ^*−/−*^ and TGN‐020 groups than in the ICH group (*p* < 0.0001). MFP treatment reduced iron deposition compared to the ICH group (*p* < 0.0001, Figure 2e,f).

### 4.3 Fluorescent tracing method was employed to investigate the impact of AQP4 on the clearance function of the glymphatic system following cerebral hemorrhage

#### 4.3.1 The temporal expression of fluorescent tracers in the murine brain

**FIGURE 3:**
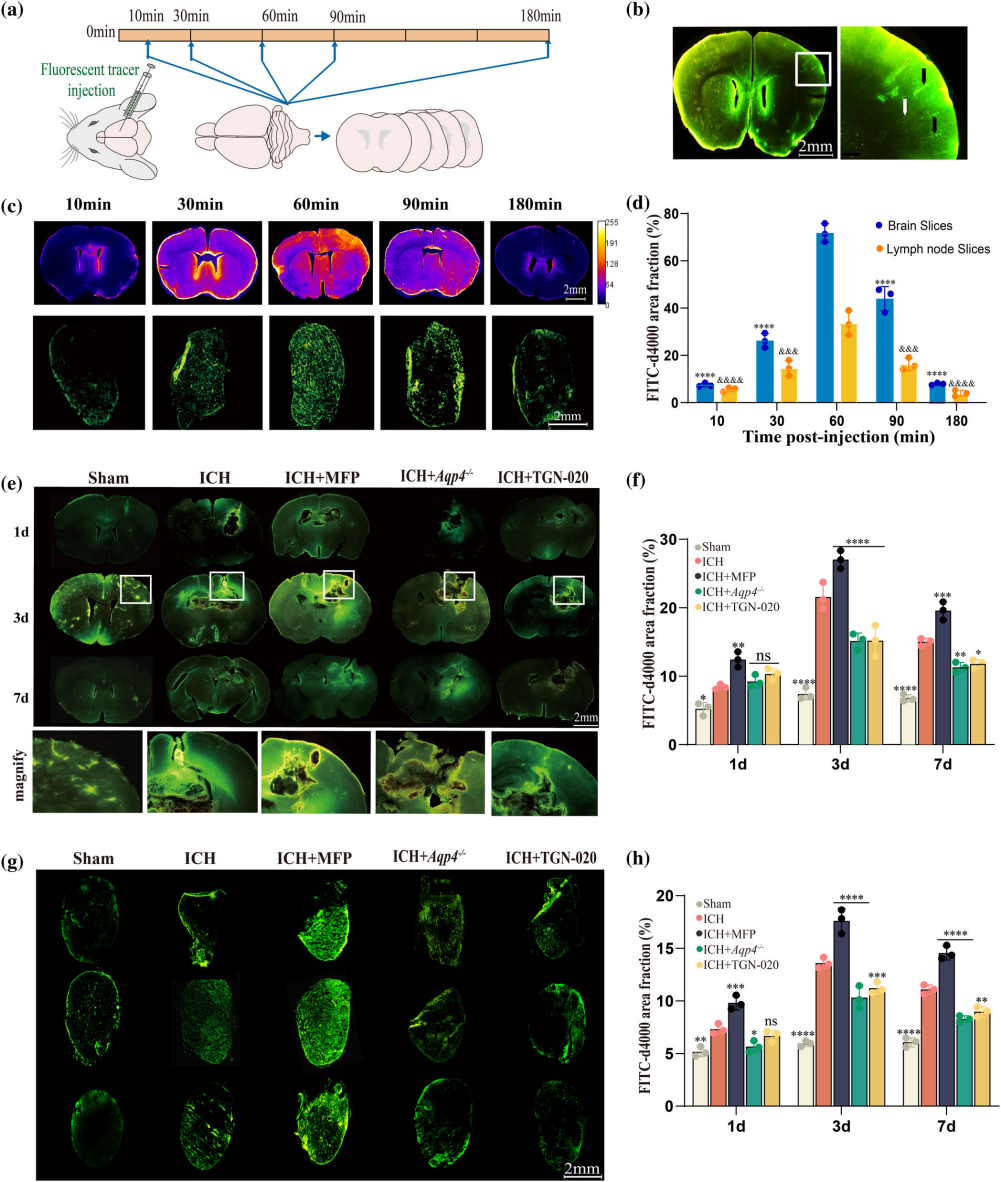
Investigating the impact of AQP4 on hematoma clearance following ICH through fluorescent tracing methodology. (a) Experimental schematic of the temporal distribution of fluorescent tracers in the mouse brain. (b) Fluorescent tracers flow through the vascular structures in the brain. (*t* = 60 min). Black arrows show tracer‐filled vessels; white arrows show untraced ones. (c) Representative images demonstrate the distribution of tracer in coronal brain sections and deep cervical lymph node sections over time. (d) Quantification reveals the permeated tracer area (*n* = 3). ****p < 0.0001 versus Time post‐injection 60 min group in brain slices. ^&&&^ *p* < 0.001/^&&&&^ *p* < 0.0001 versus time post‐injection 60 min group in lymph node slices. (e) Distribution of fluorescent tracers at different time points in various groups Fluorescent image of FITC‐d4000 distribution in brain and magnified view of the white box in Panel. Clearly visible hematoma and injection site in the image. (f) Quantification of fluorescent area proportion in coronal sections of brain tissue (*n* = 3). *p < 0.05/**p < 0.01/***p < 0.001/****p < 0.0001 versus ICH group at the same time point. ns: no significant versus ICH group at the same time point. (g) Fluorescent tracer distribution and quantification (h) in deep cervical lymph nodes (*n* = 3). *p < 0.05/**p < 0.01/***p < 0.001/****p < 0.0001 versus ICH group at the same time point. ns: No significant versus ICH group at the same time point. White scale bar:2 mm. AQP4, aquaporin‐4; ICH, intracerebral hemorrhage.

To simulate brain metabolic byproduct clearance, we injected the FITC‐d4000 tracer into the right basal ganglia of normal mice. Changes in the fluorescence distribution were monitored over time to assess glymphatic system function (Figure 3a). Post‐tracer injection, distinct vascular structures appeared in the cortical region (Figure 3b). The fluorescence permeation area was observed at different time points (10, 30, 60, 90, 180 min) in coronal sections. The results showed a significant increase in the permeation area within the first 60 min post‐injection. At 10 min, the tracer was concentrated around the ventricles, with noticeable meningeal accumulation by 30 min. Peak fluorescence was at 60 min, followed by a decreasing trend. The fluorescence permeation areas at 10, 90, and 180 min were statistically different from that at 60 min (*p* < 0.0001, Figure 3c,d). Simultaneously, the deep cervical lymph nodes were extracted from the mice at the corresponding time points for observation. Upon injecting the FITC‐d4000 tracer into the right basal ganglia, a significant accumulation was noted in the deep cervical lymph nodes, indicating extracranial circulation through these nodes. The assessment of fluorescence permeation area changes in the lymph nodes showed that FITC‐d4000 peaked at 60 min. Comparing the 10‐min(*p* < 0.0001), 30‐min (*p* = 0.0002), 90‐min (*p* = 0.0003), and 180‐min (*p* < 0.0001) groups with the 60‐min group, statistically significant differences were found, with the 60‐minute group having a higher fluorescence permeation area (Figure 3c,d). In this experiment, we observed that the fluorescent tracer rapidly permeated the entire brain upon entry, with a significant increase in fluorescence intensity at the vascular sites. Subsequently, it exited through the lymphatic system, evident in the deep cervical lymph nodes.

#### 4.3.2 The influence of AQP4 intervention on the functionality of the glymphatic system after ICH

We employed a fluorescent tracer to simulate brain waste clearance, the tracer area reflects the brain's clearance function. We investigated the distribution of fluorescent tracers in different groups. We injected the tracers into the site of ICH in mice and collected brain tissue specimens for frozen sectioning at 60 min post‐injection. We focused on coronal sections of brain tissue with hemorrhagic lesions to gain a better understanding of the hematoma clearance dynamics. (Figure 3e). Brain tissue slices at 1, 3, and 7 days post‐ICH showed a significant increase in fluorescent tracer area at the same time points compared to the Sham group (Figure 3e,f). At 1 day post‐ICH, the ICH group exhibited a larger area of fluorescent distribution compared to the Sham group (*p* = 0.0154), with a broader distribution observed following MFP intervention (*p* = 0.0016). However, the groups receiving TGN‐020 (*p* = 0.9103) intervention or *Aqp4* gene knockout (*p* = 0.2801) showed no significant differences compared to the ICH group (Figure 3e,f). At 3 days, ICH + MFP exhibited increased fluorescence, while ICH + *Aqp4* ^*−/−*^ and ICH + TGN‐020 exhibited decreased fluorescence compared to the ICH group (*p* < 0.0001). Similar trends were observed at 7 days, with ICH + MFP showing an increase (*p* = 0.0003), and *Aqp4* ^*−/−*^ (*p* = 0.0138), and TGN‐020(*p* = 0.0036) intervention groups exhibiting a decrease compared to the ICH group (Figure 3e,f). Frozen sections of deep cervical lymph nodes also showed tracer accumulation (Figure 3g). In the 1, 3, and 7‐day groups, the ICH group exhibited significantly increased fluorescence areas compared to the Sham group (*p* = 0.0014, *p* < 0.0001, and *p* < 0.0001, respectively). At 3 and 7 days, the ICH + MFP group showed a significant increase compared to the ICH group (*p* < 0.0001, *p* < 0.0001), while the ICH + *Aqp4* ^*−/−*^ group (*p* < 0.0001, *p* < 0.0001) and the ICH + TGN‐020 group (*p* = 0.0004, *p* = 0.0018) exhibited significant decreases (Figure 3h).

### 4.4 Quantifying the impact of AQP4 on the glymphatic system in mice after ICH was achieved through the application of MRI methodology

In the prior experiment, AQP4 intervention significantly affected hematoma clearance and neurological function scores in murine brain slices. Therefore, utilizing dynamic MRI to assess the impact of various interventions on post‐ICH hematoma and edema is crucial.

**FIGURE 4:**
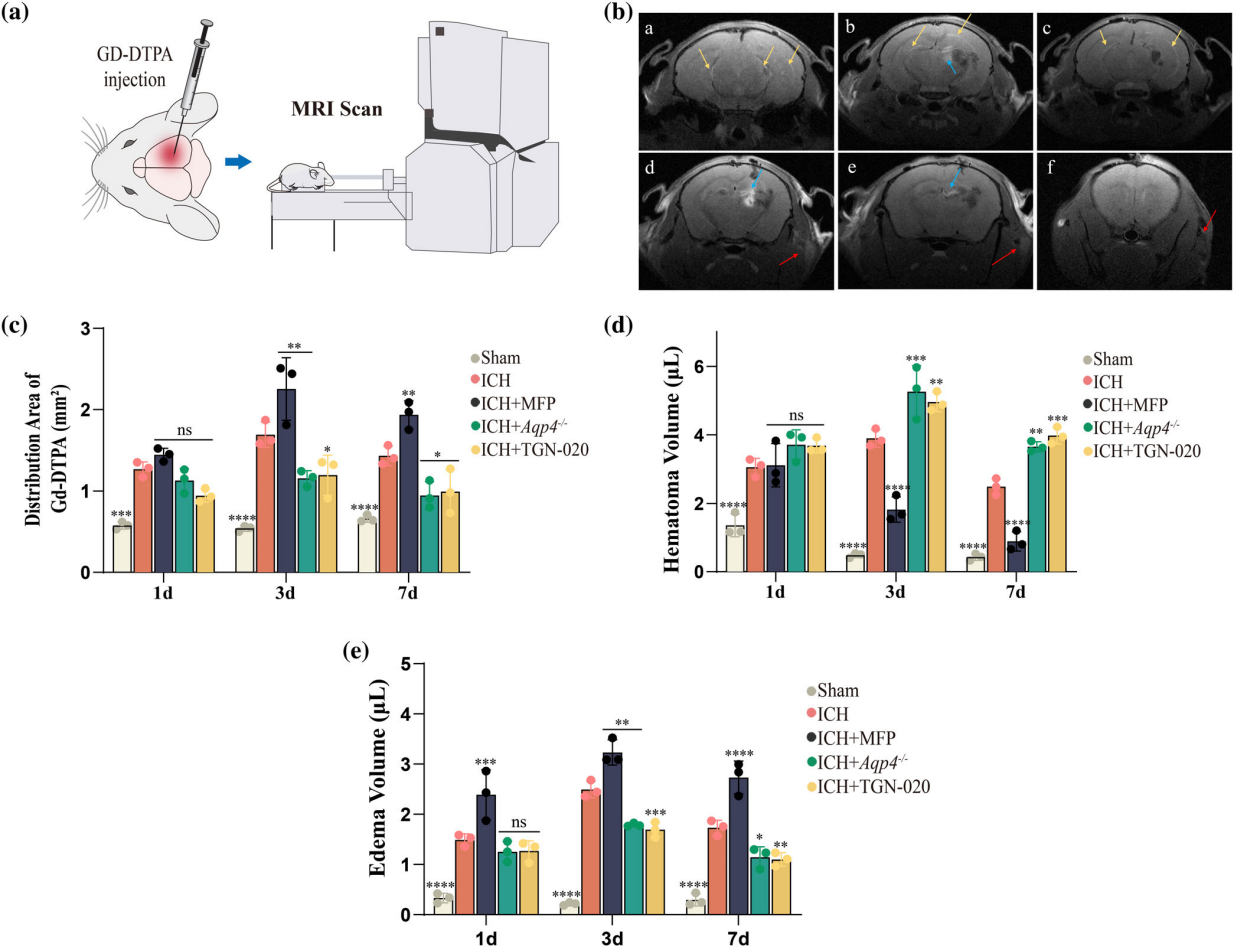
Investigating the role of AQP4 in hematoma clearance after ICH through MRI scanning. (a) Experimental schematic. (b) Diffusion distribution of contrast agent (GD‐DTPA) under continuous scanning, with labeled images a‐f representing consecutive coronal sections. Yellow, blue, and red arrows indicate contrast agents distribution in ventricles, brain parenchyma, and cervical vessels/lymph nodes, respectively. (c) The distribution of contrast agent Gd‐DTPA was assessed using MRI at 1, 3, and 7 days post‐cerebral hemorrhage (T1WI sequences). Quantification of contrast agent diffusion area in brain (*n* = 3). (d) The volume of cerebral hemorrhage was evaluated using MRI at 1, 3, and 7 days post‐ICH (SWI sequences). Quantification of hematoma volume in brain.(n = 3) (e) The volume of edema was evaluated using MRI at 1, 3, and 7 days post‐ICH (T2WI sequences). Quantification of edema volume in brain. (*n* = 3). *p < 0.05/ **p < 0.01/***p < 0.001/****p < 0.0001 versus ICH group at the same time point. ns: No significant versus ICH group at the same time point. AQP4, aquaporin‐4; ICH, intracerebral hemorrhage.

Using the same grouping as in the previous experiment, we administered Gd‐DTPA directly into the ICH site in mice at 1, 3, and 7 days post‐ICH. MRI scans were conducted 6 h after intracranial Gd‐DTPA injection to observe glymphatic system‐mediated hematoma clearance pathways post‐ICH, and to assess the impact of AQP4t on this system's functionality in hematoma clearance (Figure 4a). Gd‐DTPA diffusion in cerebral parenchyma appeared as high signals on TIWI MRI. Grayscale values represented diffusion area. In the ICH group images, high signals were observed in the ventricles, cerebral parenchyma, and submandibular region, corresponding to the cervical vessels and adjacent lymph nodes (Figure 4b).

Compared to the Sham group, the ICH group exhibited significantly larger Gd‐DTPA diffusion areas in the live mouse brain at 1 (*p* = 0.0002), 3 (*p* < 0.0001), and 7 days (*p* < 0.0001) post‐ICH, indicating enhanced lymphatic system functionality after ICH (Figures 4c and S1a). This is consistent with the results of our fluorescent tracer experiment. Further comparison revealed that at 3 and 7 days post‐ICH, the diffusion area in the ICH + MFP group increased compared to that in the ICH group (Figure 4c, *p* = 0.0029; *p* = 0.0083). In contrast, *Aqp4* ^*−/−*^ and TGN‐020 administration resulted in smaller contrast agent diffusion areas at 3 days (*p* = 0.0048;*p* = 0.01) and 7 (*p* = 0.0127; *p* = 0.0289) days post‐ICH, indicating regulated lymphatic system functionality (Figures 4c and S1a). These findings underscore the impact of AQP4 regulation on the lymphatic system, influencing contrast agent diffusion areas.

In this study, we confirmed enhanced glymphatic system functionality post‐ICH in live mice. AQP4 agonists further augmented glymphatic function, whereas inhibitors and gene knockout diminished it. Our analysis focused on understanding how these glymphatic system changes impacted hematoma and edema in the mouse brain at different time points across the experimental groups. Hematoma analysis post‐ICH used the SWI sequence (Figures 4d and S1b). At 1 day post‐ICH, cerebral hematoma volumes were comparable across the groups, except for the sham surgery group.

*Aqp4* ^*−/−*^(*p* = 0.0005) and TGN‐020 (*p* = 0.0082) groups showed larger hematoma volumes at 3 days post‐ICH compared to ICH, while the MFP (*p* < 0.0001) intervention resulted in a smaller, statistically significant hematoma volume (Figures 4d and S1b). At 7 days post‐ICH, a consistent pattern emerged: compared to the ICH group, the ICH + MFP group exhibited a reduced hematoma volume (*p* < 0.0001), whereas both the *Aqp4* ^−/−^ (*p* = 0.0032) and TGN‐020 (*p* = 0.0002) groups demonstrated an increase in hematoma volume (Figures 4d and S1b).

Perihematomal edema analysis using T2WI sequences in mouse MRI showed distinct outcomes. At 1 day post‐ICH, the volume of edema in the ICH group significantly increased compared to the Sham group (*p* < 0.0001, Figures 4e and S1c). The edema volume was even larger in the MFP intervention group compared to the ICH group (*p* = 0.0001), while there were no significant differences observed between the *Aqp4* ^−/−^ and TGN‐020 groups compared to the ICH group (*p* > 0.05, Figures 4e and S1c). Comparison at day 3 post‐ICH revealed that, relative to the natural ICH progression, the MFP intervention group exhibited a larger edema volume (*p* = 0.0016), whereas the TGN‐020 (*p* = 0.0028) and *Aqp4* gene knockout (*p* = 0.0006) groups displayed smaller volumes (Figures 4e and S1c). At 7 days post‐ICH, during the recovery phase, edema volumes decreased, aligning with the trend observed at 3 days (ICH vs. MFP: *p* < 0.0001; ICH vs. *Aqp4* ^−/−^:*p* = 0.0159; ICH vs. TGN‐020:*p* = 0.0083. Figures 4e and S1c).

### 4.5 Cellular localization and polarized expression of AQP4

**FIGURE 5:**
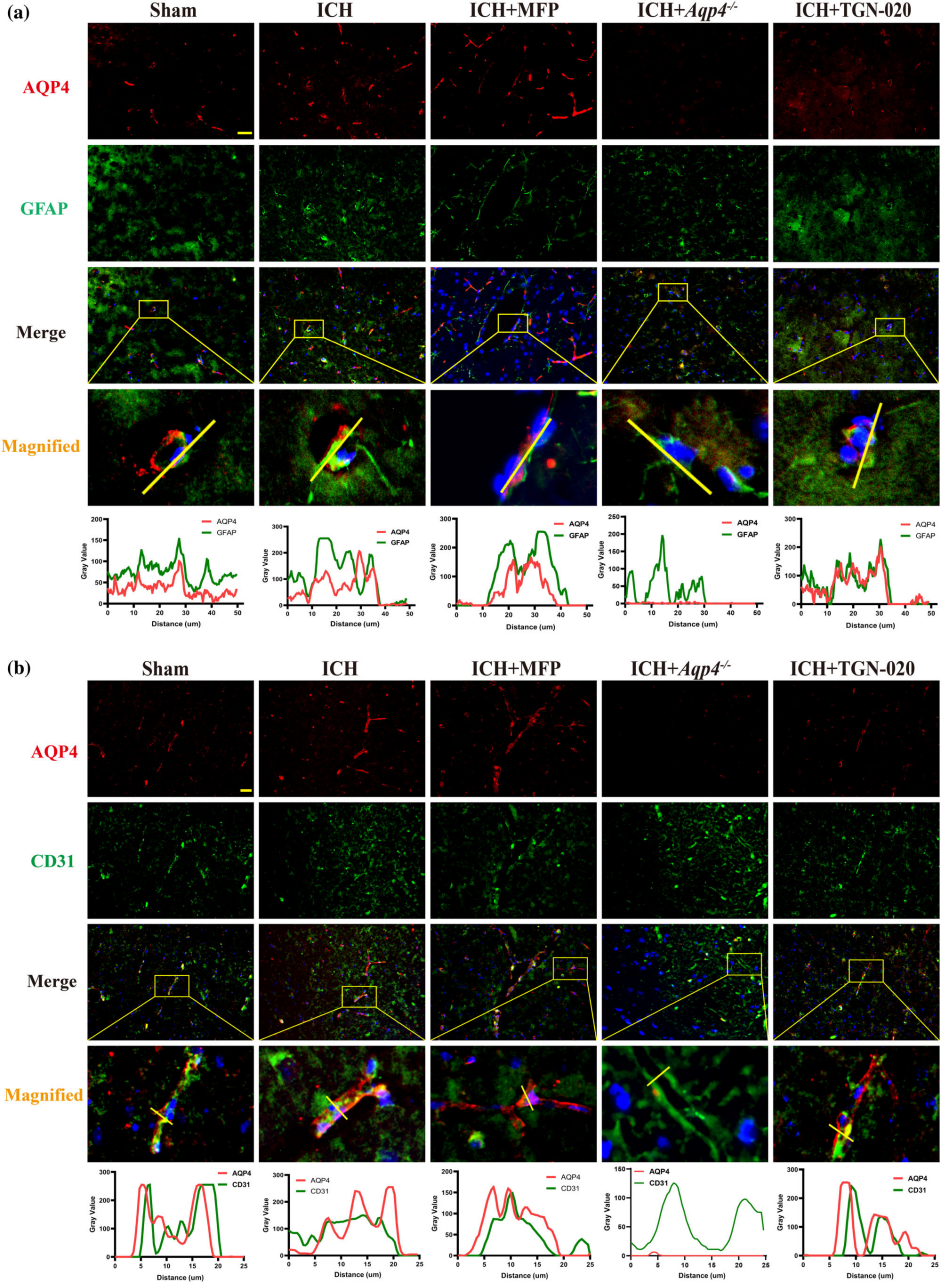
The cellular localization of AQP4. (a) Immunofluorescent double‐label images of AQP4 and GFAP for each group, along with co‐localization analysis. AQP4 is marked with red fluorescence, GFAP with green fluorescence and “Merge” represents the overlay of the first three images. “Magnified” shows locally enlarged images. (b) Polarized distribution of AQP4 observed by calculating co‐localization with CD31. AQP4 is marked with red fluorescence, CD31 with green fluorescence. CD31 is an adhesion molecule for platelets and endothelial cells, serving as a marker for blood vessels. Yellow scale bar:50 μm. AQP4, aquaporin‐4; ICH, intracerebral hemorrhage.

We explored AQP4 cellular localization via immunofluorescence co‐staining with GFAP, an astrocytic marker. Comparative analysis revealed increased AQP4 expression on astrocytes in the ICH group compared to that in the Sham group. Following MFP intervention, AQP4 expression further increased compared to the ICH group, while AQP4 expression was suppressed in the *Aqp4−/−* and TGN‐020 groups (Figure 5a). Co‐localization results showed a partial overlap in AQP4 and GFAP expression with significant differences in intensity. Except for MFP, all groups exhibited a similar increasing and decreasing trend in AQP4 expression with astrocytic GFAP (Figure 5a).

We observed polarized expression of AQP4 around blood vessels, as evidenced by the immunofluorescence results around CD31‐labeled vessels. Comparative analysis revealed a more pronounced polarized distribution of AQP4 around blood vessels in the ICH and MFP groups, particularly in the MFP intervention group where AQP4 polarization increased. In contrast, reduced expression and polarization of AQP4 around the blood vessels were observed in the *Aqp4* ^*−/−*^ and TGN‐020 groups. The colocalization results of AQP4 with CD31 displayed a consistent expression trend on both sides of the lumen in cross‐sectional views of the blood vessels. Notably, AQP4 polarization increased with CD31 expression in the MFP group compared to the Sham and ICH groups, while polarization diminished in the *Aqp4* ^*−/−*^ and TGN‐020 groups (Figure 5b).

## 5 DISCUSSION

The glymphatic system primarily describes the convective flow of CSF from the cortical subarachnoid space through perivascular spaces into the brain parenchyma, with subsequent clearance along venous pathways (Iliff et al., 2012). Due to the difficulty in anatomically defining this system, it remains controversial. Mohammad et al., using a one‐dimensional model, analyzed fluid flow in terms of pressure and resistance, suggesting that rapid flow within the perivascular spaces is unlikely (Faghih & Sharp, 2018). However, increasing evidence supports the existence of the glymphatic pathway and the mediating role of AQP4 (Benveniste et al., 2021; Hablitz et al., 2020; K et al., 2022). Given AQP4's hydrophilic properties, this system cannot be adequately evaluated using only one‐dimensional models and fluid pressure dynamics.

In experiments using fluorescent tracers and MRI contrast agents to assess the glymphatic system, the ICH group exhibited significantly larger fluorescence tracer/contrast agent areas than the Sham group. WB analysis revealed increased AQP4 protein levels post‐CH, indicating an enhanced clearance function within the glymphatic system. Fluorescence tracing at 3 and 7 days revealed inter‐group differences in hematoma area, which was negatively correlated with cervical lymph node fluorescence and positively correlated with neurological deficit scores. MRI‐based calculations of hematoma volume supported these conclusions. These results suggest a direct correlation between glymphatic system clearance function and hematoma resolution. Faster clearance corresponded to diminished hematoma sizes and improved neurological outcomes. In the ICH model using the AQP4 inhibitor and *Aqp4* ^*−/−*^ mice, reduced AQP4 expression resulted in an amplified hematoma area and volume, exacerbated neurological deficits, and impeded hematoma clearance. MFP administration upregulated AQP4 expression, enhanced glymphatic system function, and facilitated hematoma clearance. This experimental validation underscores AQP4's pivotal regulatory role in hematoma clearance.

Hematoma, a critical factor in post‐ICH brain injury, induces structural damage and inflammation. Effective clearance is essential for alleviating post‐bleeding damage (Cordonnier et al., 2018). Dysfunction of the glymphatic system, particularly involving AQP4, is associated with various neurological disorders characterized by pathological solute accumulation, such as Aβ and Tau clearance in AD (Plog & Nedergaard, 2018). In a traumatic brain injury (TBI) mouse model, *Aqp4* knockout impairs solute clearance (Wang, Ding, et al., 2017). However, its role in the clearance of intraparenchymal hematoma clearance remains unclear. Our study revealed that upregulation of AQP4 mitigates post‐ICH neurological damage, BBB disruption, and cerebral Hb accumulation, while inhibiting AQP4 leads to opposing outcomes.

AQP4 is part of the aquaporin family and is expressed in ependymal cells of the brain ventricles, astrocytes, the Purkinje layer of the cerebellum, the hypothalamus, and other regions (Deffner et al., 2022). It is the primary facilitator of transmembrane water transport in the brain, reducing the driving force for fluid transmembrane transport (Nagelhus & Ottersen, 2013; Verkman et al., 2017). AQP4 expression is 10 times higher in astrocytic endfeet than in non‐endfeet regions, a phenomenon known as AQP4 polarization (Nagelhus & Ottersen, 2013). Polarized AQP4 facilitates CSF entry into brain ISF and exchange with it (Xin et al., 2023), playing a crucial role in waste clearance through the glymphatic system (Mestre et al., 2020). Interstitial solutes and brain metabolic waste flow into perivascular spaces, eventually reaching lymphatic vessels in the meninges and cranial nerve sheaths, and are cleared through cervical lymph nodes (Lv et al., 2021). Some studies suggest that waste is also transferred from the brain to the cervical lymph nodes via the cribriform plate and olfactory nerves (Cserr & Knopf, 1992). To further investigate AQP4 function, AQP4 knockout mice have been used, which exhibit perivascular swelling of astrocytes and a resulting reduction in extracellular space in the brain parenchyma (Igarashi et al., 2013). Iliff et al. demonstrated that AQP4 knockout mice had approximately 70% reduced glymphatic clearance compared to wild‐type mice, resulting in a 55% reduction in amyloid‐β clearance in the brain (Iliff et al., 2012). Moreover, treatment with the AQP4 inhibitor TGN‐020 significantly impaired glymphatic CSF‐ISF exchange and tau protein clearance (Harrison et al., 2020). These studies highlight the critical role of the glymphatic system in brain waste clearance and the importance of AQP4.

In our study, both pharmacological inhibition and genetic knockout of AQP4 reduced the distribution of tracers and contrast agents, while treatment with an AQP4 agonist increased their distribution, ultimately influencing hematoma clearance and neurological outcomes. This confirmed the significance of AQP4 in the hematoma clearance process following ICH. In the immunofluorescence experiments, all groups, except the control group, exhibited increased perivascular astrocytes around the hematoma, accompanied by elevated AQP4 expression and polarization. Co‐localization analysis of AQP4 and CD31 indicated that the AQP4 agonist, MFP, promoted polarization, while the AQP4 inhibitor had the opposite effect. However, we also observed that over time, the hematoma in the AQP4 knockout or inhibition groups gradually decreased, and the difference from other groups diminished. We speculate this is due to compensatory mechanisms, such as vascular clearance pathways and increased phagocytic activity. Studies have shown that localized cerebral blood flow increases in AQP4 knockout or inhibited mice, supporting this hypothesis (Igarashi et al., 2013).

As a key water channel protein, AQP4 occupies approximately 50% of the surface area of astrocytic endfeet facing capillaries, forming a low‐resistance pathway for water movement and regulating fluid exchange in the CNS (Nielsen et al., 1997). After ICH, factors such as thrombin, Hb degradation products, inflammatory mediators, interleukins, and metalloproteinases collectively promote edema formation (Z et al., 2009). AQP4 upregulation further increases brain water content, but MRI detection shows no worsening of brain edema, and neurological function scores improve. We believe that the elevation of AQP4 enhances CSF‐ISF exchange, improves fluid drainage, increases overall fluid transport, and reduces edema formation, thereby accelerating hematoma clearance. This is consistent with the observations by Hussain et al. in their study on edema formation after TBI (Hussain et al., 2023). The research confirms that AQP4 mediates bidirectional water flux, facilitating water influx during cytotoxic edema formation and promoting water efflux during vasogenic edema resolution (Vella et al., 2015). In the acute phase, AQP4 promotes CSF and ISF exchange, increasing brain water content without causing additional brain edema, thus aiding in long‐term edema resolution (Fukuda & Badaut, 2013). These findings are consistent with our experimental results.

In this study, we present the first comprehensive analysis of glymphatic system functional changes in an ICH mouse model. Following cerebral hemorrhage, we observed an upregulation in both AQP4 expression and polarization. Pharmacological interventions, including AQP4 inhibition and genetic knockout, significantly influenced post‐cerebral hemorrhage hematoma clearance, suggesting AQP4 as a potential therapeutic target for treating cerebral hemorrhage. However, hematoma clearance post‐ICH involves the glymphatic system, astrocytes, meningeal lymphatic system, and neurovascular units (Da Mesquita et al., 2021; Liu et al., 2021). Therefore, we need to integrate the glymphatic system into a broader network of brain clearance for study. Additionally, the intricate interactions between AQP4 and brain edema are of interest and represent a future research directions.

## 6 CONCLUSION

The glymphatic system plays a crucial role in hematoma clearance following ICH. Upregulation of AQP4 improves glymphatic system function, thereby facilitating hematoma clearance, and reducing BBB permeability, neuronal death rates, and iron deposition. AQP4 overexpression leads to improvements in neurological function and the restoration of damaged brain tissue structure after cerebral hemorrhage.

## AUTHOR CONTRIBUTIONS

All authors listed have made a substantial, direct, and intellectual contribution to the work, and approved it for publication. Conceived and designed the experiments by Gaiqing Wang; Performed the experiments by Wenchao Chen, Chuntian Liang, Shasha Peng, and Shuangjin Bao; Analyzed and interpreted the data by Wenchao Chen and Chuntian Liang; Contributed reagents, materials, analysis tools, or data by Gaiqing Wang, Fang Xue, Xia Lian, and Yinghong Liu; Wrote and edited the article by Wenchao Chen and Gaiqing Wang.

## FUNDING INFORMATION

This work was supported by Region Program of Natural Science Foundation of China (NO: 82160237); Key Research and Development Program in Hainan Province (NO: ZDYF2023 SHFZ104); General Program of Natural Science Foundation of Hainan Province (NO: 822MS210); Sanya Science and Technology Innovation Special Project (NO: 2022KJCX24). The funders had no role in study design, data collection and analysis, decision to publish, or preparation of the manuscript.

## CONFLICT OF INTEREST STATEMENT

The authors declare that they have no affiliations with or involvement in any organization or entity with any financial interest in the subject matter or materials discussed in this manuscript.

## Supporting information


**Data S1:** Supporting Information.

## Data Availability

The data that support the findings of this study are available upon request from the corresponding author. Any additional information or data required to reproduce the results can be provided by contacting the corresponding author at wanggq08@163.com.
