# Supplementary material for: Aquaporin‐4 activation facilitates glymphatic system function and hematoma clearance post‐intracerebral hemorrhage
Source: Glia. 2024 Nov 12;73(2):368–80. doi: 10.1002/glia.24639 (PMC11662979; doi:10.1002/glia.24639)
Supplement: Supplementary file 1 — Data S1: Supporting Information. [file GLIA-73-368-s001.docx]

**SUPPLEMENTAL MATERIAL**

**The role of AQP4 in regulating glymphatic system in experimental intracerebral hemorrhage**

Wenchao Chen^1^, Chuntian Liang^1,2^, Shasha Peng^3,4^, Shuangjin Bao^5,6^, Fang Xue^1^, Xia Lian^1^, Yinghong Liu^1^, Gaiqing Wang^1,2*^

^1^ Department of Neurology, Second Hospital of Shanxi Medical University, Shanxi Medical University, 030001 Taiyuan, Shanxi, China.

^2^ Department of Neurology, Sanya Central Hospital (Hainan Third People’s Hospital), Hainan Medical University, 572000 Sanya, Hainan, China.

^3^ Department of Pharmacology, School of Basical Medical Sciences, Shanxi Medical University, 030001 Taiyuan, China.

^4^ Department of Pharmacy, Sanya Central Hospital (Hainan Third People’s Hospital), Hainan Medical University, 572000 Sanya, China.

^5^ Department of Pathology and Pathophysiology, Basic Medical College, Shanxi Medical University, 030000 Taiyuan, China.

^6^ Department of Pathology, West China Fourth Hospital, 610041 Chengdu, China.

***Corresponding author:** Gaiqing Wang, Email: [wanggq08@163.com](mailto:wanggq08@163.com)

**Table of Contents**

**1 Supplemental Methods** **3**

**1.1 Animal materials** 3

**1.2 Experimental design** 3

**1.3 ICH models** 3

**1.4 Neurobehavior tests** 3

**1.5 Measurement of Brain Water Content and Blood-Brain Barrier (BBB)Permeability** 3

**1.6 Hemoglobin (Hb) Assay** 4

**1.7 Neuronal degeneration was assessed using Fluoro-Jade C (FJC) staining**4

**1.8 Iron staining** 4

**1.9 Nissl Staining** 4

**1.10 Fluorescent tracing method** 4

**1.11 Dynamic Multi-Modal Magnetic Resonance Imaging(MRI) method**5

- 1. **AQP4 expression** 5
  2. **Cellular localization of AQP4 and fluorescence double-labeling Immunofluorescence**6

**2 Supplemental Figures** **7**

**3 Supplemental Tables** **7**

**1** **Supplemental Methods**

**1.1 Animal materials**

Male adult C57 BL/6 mice (6-8 weeks old, ~25g), including 182 wild-type mice from the Animal Experimental Center of Shanxi Medical University (Taiyuan, China) and 38  *Aqp4 gene knockout* *(Aqp4^-/-)^* mice purchased from Cyagen Model Biological Research Center (Suzhou, China), were utilized. Mice were housed under suitable temperature and humidity conditions with ad libitum access to food and water. A one-week acclimatization period preceded the experiments.

**1.2 Experimental design**

The experimental mice were randomly assigned to the following groups: Sham surgery (Sham, 5%DMSO, twice daily, o.g.), Intracerebral Hemorrhage (ICH, 5%DMSO, twice daily,o.g.), ICH mice treated with the AQP4 agonist mifepristone (ICH+MFP, 35mg/kg/d, twice daily, o.g.), ICH mice treated with AQP4 inhibitor TGN-020 (ICH+TGN-020, 100mg/kg, once daily, i.p.), and *Aqp4^-/-^* mice with ICH (ICH+ *Aqp4^-/-^*). Mifepristone was purchased from Shanghai Yuanye Bio-Technology Co., Ltd.( Cat# S25748), "TGN-020 was purchased from TargetMol(Cat# 51987-99-6). The first drug intervention was administered 2 hours after the intracerebral hemorrhage surgery in mice, ensuring that the mice were fully awake from anesthesia at the time of administration.

**1.3 ICH models**

As prior experiments^7,9^, mice were anesthetized by intraperitoneal injection of pentobarbital (10 mg/kg) and ketamine (100 mg/kg). Mice were positioned prone in a stereotaxic frame following skin preparation. A midline incision at the exposed mouse head's center revealed coordinates 0.9 mm posterior to the bregma and 1.5 mm lateral to the midline. Using a rosyringmice, 0.65 μL of type IV collagenase (2 mg/mL, Solarbio, Cat# BS165) was injected into the deep brain, halting at 3.6 mm depth. After a 15-min dwell, the needle was withdrawn, sealing with bone wax. The scalp was sutured. Sham mice received saline. Post-op, mice recovered on a 37°C heating pad, with vital sign monitoring. The Rosenberg system assessed the ICH model, deeming it satisfactory at a score of 1 or higher.

**1.4 Neurobehavior tests**

After mouse modeling, neurological deficit scoring on days 1, 3, and 7 used the Modified Garcia Scale. Scored in a double-blind manner by collaborators, it includes six items: bilateral limb activity, spontaneous activity, mesh wall climbing, extension of forelimbs, whisker response on one side, and trunk touch response. Each item is scored from 0 to 3 based on the strength of mouse neurological function, with 0 being the weakest and 3 being the strongest, resulting in a minimum score of 3 and a maximum score of 18. A lower score indicates more severe neurological functional impairment. See Table S1 for details(Table.S1).

**1.5 Measurement of Brain Water Content and Blood-Brain Barrier (BBB) Permeability**

Two hours prior to euthanasia, mice received an intravenous injection of 2% Eans Blue (EB, 0.004 ml/g, Sigma, Cat# E2129) via the tail vein. Following deep anesthesia, cardiac perfusion was performed using physiological saline, and then the brain tissue was extracted. Harvest the ipsilateral hemisphere with hematoma. Cut 1.5mm coronal slices anterior and posterior to the needle track created for modeling. These slices are used respectively for assessing BBB permeability and brain water content.

For BBB permeability, brain tissue was weighed, homogenized in 600 μL saline, and centrifuged at 4°C. The resulting 500 μL supernatant was mixed with an equal volume of 50% trichloroacetic acid at 37°C, incubated for 72 hours. OD value at 610nm was determined using a UV spectrophotometer, and EB brain content (μg/g brain tissue) was calculated using the formula: EB concentration × total solvent volume (mL) ÷ brain wet weight (g).

For brain water content, specimens were weighed for wet weight, then placed in a 100°C drying oven for 48 hours to obtain dry weight. Brain water content (%) was calculated using the formula: (wet weight - dry weight) / wet weight.

**1.6** **Hemoglobin（Hb）Assay**

After anesthesia, mice underwent cardiac perfusion with saline. The bleeding hemisphere of the brain was retained, and coronal slices 1.5mm anterior to the needle track were taken for subsequent experiments. After centrifugation, 200 μL of the resulting supernatant was collected. Each group's 200 μL supernatant was mixed with 800 μL Van Kampen and Zijlstar Solution(Xinfan Biotechnology, Cat# F0112) at a 1:4 ratio for 5 minutes at room temperature. The OD value at 540nm was measured using a UV spectrophotometer, and calculations used the hemoglobin (Hb) standard curve. Hb brain content (μg): Hb concentration × total solvent volume (mL).

**1.7** **Neuronal degeneration was assessed using Fluoro-Jade C (FJC) staining.**

After anesthesia, mice underwent cardiac perfusion with 4% paraformaldehyde. Harvested brain tissue was fixed in 4% paraformaldehyde for 24 hours. Dehydration in 20% and 30% sucrose solutions, followed by coronal sectioning at 7 μm thickness using a cryostat. Frozen sections were stained using the FJC staining kit (Solarbio, Cat# G3262) following the provided protocol. Incubate the sections with reagent A for 5 minutes. Then, immerse them in 70% ethanol for 2 minutes followed by a 5-minute rinse with distilled water. Subsequently, treat the sections with reagent B for 10 minutes, rinse with distilled water for 2 minutes, and then incubate in darkness at room temperature for 10 minutes with reagent C. After three rinses with distilled water, immerse the sections in xylene for 1 minute, mount with neutral resin, and observe . The percentage of degenerated neurons in the entire slice field of view was calculated using ImageJ, and comparisons were made between groups.

**1.8** **Iron staining**

The slice preparation method was the same as that for FJC staining. Frozen sections were initially stained with a mixture of equal volumes of Perls reagent box(Solarbio, Cat# G1424) A1 solution (2% potassium ferrocyanide) and A2 solution (2% hydrochloric acid) for 30 minutes. B solution (neutral red) was added dropwise for a 20-second stain, followed by immersion in deionized water for 2-3 seconds. Ethanol dehydration (70%, 85%, 95%, 100%) and neutral resin sealing preceded microscopic observation，calculate the percentage of the positive area in iron staining.

**1.9 Nissl Staining**

The slice preparation method was the same as that for FJC staining. Frozen sections were fixed in 4% paraformaldehyde for 10 minutes, washed for 2 minutes in distilled water. Nissl staining for 30 minutes, two washes in distilled water, 2-minute dehydration in 95% ethanol, 5-minute xylene transparency, and neutral resin sealing.

**1.10 Fluorescent tracing method**

**Drug Administration**

The FITC-d4000 (Chongqing Yucy Medicine, Cat# YS-DE720) was prepared as a 1% solution using artificial cerebrospinal fluid, shaken thoroughly, and maintained in the dark throughout the process. The solution was freshly prepared for immediate use.

**Fluorescent tracer injection**

Anesthetize mice and position them prone on a stereotaxic apparatus. Slowly inject 10 μL FITC-d4000 into the brain using a microsyringe (following the same procedure and location as during ICH model creation).

**Fluorescence tracer images acquired**

The method for obtaining slices is the same as for FJC, frozen sections were prepared, with the distinction that the brain coronal sections were cut to a thickness of 100 μm. Similar procedures were applied to the deep cervical lymph nodes, with a section thickness of 10 μm. After obtaining frozen sections, they were sealed with anti-fluorescence quenching mounting medium. Fluorescence microscopy was used for image capture, Autopano Giga 3.7 was employed for synthesis, and Image J 8.0 for analysis.

**1.11 Dynamic Multi-Modal Magnetic Resonance Imaging(MRI) method**

**MRI image acquisition**

6 hours before MRI scanning , 0.5 μL of the contrast agent Gd-DTPA (10 mmol/L) was injected into the mice's brains using the same method as the fluorescent tracer. Based on prior team experiments , mice underwent head MRI scans on days 1, 3, and 7 post-intracerebral hemorrhage using a 3.0 Tesla clinical scanner (DISCOVERY*MR750W). The MRI protocol included T1-weighted imaging (T1WI), susceptibility-weighted imaging (SWI), fluid-attenuated inversion recovery sequence (T2-Flair), and T2-weighted imaging (T2WI). The scanning parameters are as follows: (1) T1WI sequence: TR (repetition time): approximately 494ms, TE (echo time): approximately 85ms, slice thickness: 1.5mm, FOV (field of view): 6x6mm, gap: 0.2mm, matrix size: 256x256, flip angle: 111°. (2) SWI sequence: TR: approximately 250ms, TE: 45ms, slice thickness: 2mm, FOV: 6x6mm, matrix size: 256x256, flip angle: 15°. (3) T2-FLAIR: TR: 5000ms, TI: 1797ms, TE: 95ms, slice thickness: 1.5mm, FOV: 6x6mm, gap: 0.2mm, matrix size: 224x224. (4) T2WI sequence: TR: approximately 2394ms, TE: approximately 85ms, slice thickness: 1.5mm, FOV: 6x6mm, gap: 0.2mm, matrix size: 256x256.

**Calculation of the average relative gray value and distribution area**

Utilizing ImageJ software, determine the maximum grayscale value within the region of interest (ROI) on the T1-weighted scan image of the brain hemorrhage side. Subtract the grayscale value of the corresponding area on the contralateral side to obtain the average relative grayscale value of the contrast agent.

**Hematoma and edema volume calculations**

Hematoma volume was determined using SWI, and edema volume was assessed via T2WI. Calculation employed the formula V = A * B * C / 2, where V represents hematoma or total lesion volume (μL), A is the area of the largest hemorrhage layer (cm²), B is the number of hemorrhage layers, and C is the thickness of the target sequence (cm). ImageJ identified the largest hemorrhage layer area as the ROI. Edema volume was calculated by subtracting SWI-measured hematoma from T2WI-measured total lesion volume using the same algorithm. Measured three times, averaged.

**1.12** **AQP4 expression**

**Messenger RNA expression**

After anesthetizing the mice, cardiac perfusion with PBS was conducted, and the brain tissues from the hemorrhagic side were collected, weighing 50 mg. Total RNA was extracted using the RNAkey™ Reagent Total RNA Extraction Kit (Seven, Cat# SM129-02). Subsequently, the RNA was reverse transcribed into cDNA using the reverse transcription kit (Mei5bio, Cat# MF166-01). Real-time quantitative PCR (qPCR) was performed on the prepared cDNA using the fluorescent quantitative kit (Mei5bio, Cat# MF797-01).

The primer sequences were shown in the table(Table.S2):

The data obtained from Real-time qPCR were analyzed using the 2-^ΔΔCt^ method (Livak method) for statistical analysis.

**Protein expression**

Tissue retrieval method is identical to PCR. Total protein was extracted using RIPA lysis buffer (Seven, Cat# SW104-01), and protein concentrations were determined with the BCA assay kit (Seven, Cat# SW101-02). Each well was loaded with 30 μg of protein for electrophoresis and subsequent membrane transfer. The membranes were individually incubated overnight at 4°C with AQP4 antibody (1:2000, Proteintech Cat# 16473-1-AP, RRID:AB_2827426), and β-actin antibody (1:8000, Bioworld Technology Cat# AP0060, RRID:AB_2797445) on a shaker. The following day, membranes were washed, followed by a 1-hour room temperature incubation with a secondary anti-rabbit antibody (1:6000, Boster Biological Technology Cat# BA1054, RRID: AB_2734136). After an additional four PBST washes, membranes were developed in a gel imaging system.

- 1. **Cellular localization of AQP4 and fluorescence double-labeling Immunofluorescence**

Each group of 3 mice was subjected to cardiac perfusion with 4% paraformaldehyde (PFA), and the brain tissues were then immersed in 4% PFA fixative for 24 hours. Subsequently, the tissues were dehydrated in 20% and 30% sucrose solutions, followed by coronal sectioning at a thickness of 7 μm using a cryostat. The sections were mounted on slides and washed three times for 5 minutes each with PBS. Antigen retrieval was performed by immersing the sections in a solution of sodium citrate buffer in a pressure cooker, boiling for 15 minutes at approximately 120°C. Afterward, the sections were blocked with 5% BSA at room temperature for 60 minutes. Primary antibodies included rabbit anti-GFAP (1:200, Abcam, ab68428), mouse anti-AQP4 (1:200, Abcam Cat# ab9512, RRID:AB_307299), and goat anti-CD31 (1:50, Servicebio, GB13063 ServiceBio Cat# GB13063, RRID:AB_2927776). For dual labeling, the two primary antibodies were mixed and applied to the tissues, followed by overnight incubation at 4°C in a humidified chamber. The next day, after three 5-minute washes with PBS, the sections were incubated at room temperature in the dark for 1 hour with a mixture of secondary antibodies diluted in PBS: Cy3-conjugated goat anti-mouse IgG (1:250, (Boster, Cat# BA1031), Dylight 488-conjugated goat anti-rabbit IgG (1:250, Boster, Cat# BA1127), and Alexa Fluor 594-conjugated rabbit anti-goat IgG (1:250, Bioss, Cat# bs-0294R). Finally, the sections were coverslipped with anti-fade mounting medium containing DAPI for fluorescent microscopy analysis. Three different fields around the hematoma with good visualization were selected for imaging, and ImageJ software was used for statistical analysis.

**Quantification of AQP4 polarization and co-localization**

The polarization of AQP4 was analyzed using the Graphics function of the Image J software. Blood vessels were identified and localized using the vascular marker CD31, and a line perpendicular to the cross-section of the vessel, measuring 25 μm in length, was drawn with the vessel at its center. Within this defined region, the expression of AQP4 was quantified to represent the polarized distribution of AQP4 around blood vessels^12-14^. The co-localization analysis of AQP4 and GFAP was conducted using a similar method (line length: 50 μm).

1. **Supplemental Figures**

**
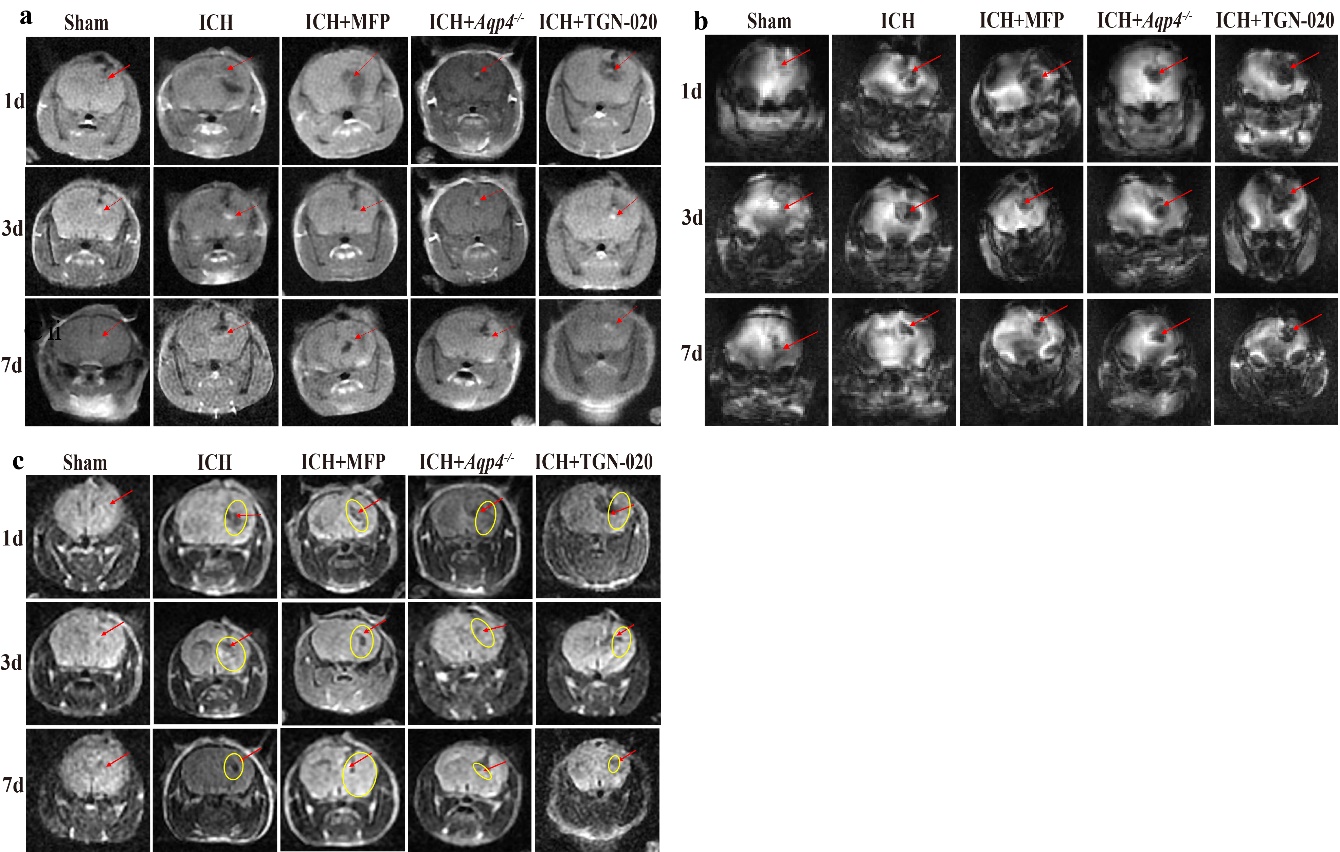
**

Figure 4 Investigating the role of AQP4 in hematoma clearance after ICH through MRI scanning. (a) Representative images of MRI (T1WI sequences). The red arrow indicates the contrast agent. (b) The volume of cerebral hemorrhage was evaluated using MRI at 1, 3, and 7 days post-ICH (SWI sequences). (c) Representative images of MRI (T2WI sequences). The red arrow indicates the edema.

**3 Supplemental Tables**

**Table S1 Modified Garcia Scale**

|  | **0** | **1** | **2** | **3** |
| --- | --- | --- | --- | --- |
| Spontaneous activity | Absence of spontaneous activity | Slight activity, inability to touch the side of the cage | Touches the side of the cage, but not all three sides | Normal activity, touches all three sides of the cage |
| Limb activity | No movement | Very slight movement of limbs | Weak movement of limbs, delayed | Symmetric movement without significant difference from baseline |
| Forelimb extension | No movement of the affected forelimb | Slight extension movement of the affected forelimb | Less extension of the affected forelimb compared to healthy side | No significant difference in forelimb extension between sides |
| Climbing | - | Falls off, unable to climb | Can climb, but with difficulty | Climbs easily, with strong grip on the cage |
| Body proprioception | - | No response to lateral push on the trunk | Slight response to lateral push on the trunk | Quick turning of the head, symmetric response to lateral push |
| Vibrissae touch response | - | No response to vibrissae touch | Slight response to vibrissae touch | Quick response to vibrissae touch, symmetric response |

**Table S2 The Primer Sequences**

| Primer | Sequences |
| --- | --- |
| AQP4 F: | 5'-GGTGGGAGGATTGGGAGTC-3' |
| AQP4 R: | 5'-GAGCGCCTATGATTGGTC-3' |
| β-actin F: | 5'-GTGCTATGTTGCTCTAGACTTCG-3' |
| β-actin R: | 5'-ATGCCACAGGATTCCATACC-3' |
